# Patterns of Selection in Anti-Malarial Immune Genes in Malaria Vectors: Evidence for Adaptive Evolution in *LRIM1* in *Anopheles arabiensis*

**DOI:** 10.1371/journal.pone.0000793

**Published:** 2007-08-29

**Authors:** Michel A. Slotman, Aristeidis Parmakelis, Jonathon C. Marshall, Parfait H. Awono-Ambene, Christophe Antonio-Nkondjo, Frederic Simard, Adalgisa Caccone, Jeffrey R. Powell

**Affiliations:** 1 Department of Ecology and Evolutionary Biology, Yale University, New Haven, Connecticut, United States of America; 2 Organisation de Coordination pour la Lutte Contre les Endémies en Afrique Centrale, Yaoundé, Cameroon; 3 Institute de Recherche pour le Développement, Yaoundé, Cameroon; University of Toronto, Canada

## Abstract

**Background:**

Co-evolution between *Plasmodium* species and its vectors may result in adaptive changes in genes that are crucial components of the vector's defense against the pathogen. By analyzing which genes show evidence of positive selection in malaria vectors, but not in closely related non-vectors, we can identify genes that are crucial for the mosquito's resistance against *Plasmodium.*

**Methodology/Principle Findings:**

We investigated genetic variation of three anti-malarial genes; *CEC1, GNBP-B1* and *LRIM1,* in both vector and non-vector species of the *Anopheles gambiae* complex. Whereas little protein differentiation was observed between species in *CEC1* and *GNBP-B1,* McDonald-Kreitman and maximum likelihood tests of positive selection show that *LRIM1* underwent adaptive evolution in a primary malaria vector; *An. arabiensis.* In particular, two adjacent codons show clear signs of adaptation by having accumulated three out of four replacement substitutions. Furthermore, our data indicate that this *LRIM1* allele has introgressed from *An. arabiensis* into the other main malaria vector *An. gambiae.*

**Conclusions/Significance:**

Although no evidence exists to link the adaptation of *LRIM1* to *P. falciparum* infection, an adaptive response of a known anti-malarial gene in a primary malaria vector is intriguing, and may suggest that this gene could play a role in *Plasmodium* resistance in *An. arabiensis.* If so, our data also predicts that *LRIM1* alleles in *An. gambiae* vary in their level of resistance against *P. falciparum*.

## Introduction

Despite ongoing control efforts during the last decades, malaria remains one of the most deadly infectious diseases. The vast majority of its burden is carried by people on the African continent, where 1 to 2 million people die annually from this disease [1]. Current malaria control efforts are hampered by the spread of insecticide and drug resistance, which has inspired research programs aimed at the development and eventual release of genetically altered mosquitoes that would be resistant to *Plasmodium falciparum* transmission. The need to identify refractory genes for this effort has focused much attention on the immune system of malaria's main vector in Africa, *An. gambiae*. The completion of the *An. gambiae* genome [2] has greatly facilitated research in this direction, and various anti-malarial immunity genes have now been identified [*e.g.* 3]–[6]. Additionally, two recent studies provided many candidate anti-malarial immune genes that are up-regulated in response to *Plasmodium* infection [7], [8].

So far little attention has been devoted to examining polymorphism of immunity genes in natural malaria vector populations [9], [10]. It is known however that molecules that are involved in interactions with pathogens, such as immune genes, are one of the major types of proteins on which positive selection has been demonstrated [11], [12]. Presumably, this is because such genes are involved in co-evolution between hosts and pathogens. In the case of malaria, if *Plasmodium* infection affects the mosquito's fitness, we may expect the accumulation of adaptive amino acid substitutions in those anti-malarial genes that are crucial in specifically limiting *Plasmodium* infection in vector species, whereas such changes should not be found in closely related species that do not transmit malaria.

That *An. gambiae* has in fact undergone an adaptive response to *P. falciparum* infection is suggested by several lines of evidence. First of all, *P. falciparum* goes through severe bottlenecks during its life cycle in this mosquito [13], demonstrating that the mosquito immune system is limiting the *Plasmodium* infection. Furthermore, *P. berghei*, which is not transmitted naturally by *An. gambiae,* produces a much higher oocyst number in *An. gambiae* than its natural pathogen *P. falciparum.* In fact, in a review of studies estimating the fitness effect of *Plasmodium* infection on *Anopheles* species, reduced fitness was observed in 10 combinations of *Plasmodium* and *Anopheles* species that do not occur naturally, whereas in 10 natural combinations, including *An. gambiae* and *P. falciparum*, no fitness effects were observed [14]. This is an indication that *Anopheles* species have evolved to limit infections of the *Plasmodium* species they come into contact with. This is corroborated by the fact that the immune response to *P. falciparum* is specific, *i.e. An. gambiae* up-regulates different genes in response to infection with *P. falciparum* vs. *P. berghei* [5], [7]. Salivary gland infection rates of *P. falciparum* in *An. gambiae* and *An. arabiensis* are typically low, ranging between 3–9% [15]. This raises the question whether selection pressures on the mosquito immune system are strong enough to result in an adaptive response to *P. falciparum* infection. However, it should be kept in mind that the data summarized above indicate that the rate and intensity of *P. falciparum* infection is likely to have been much higher when *Anopheles* mosquitoes first came into contact with this pathogen.

*An. gambiae* belongs to a complex of closely related species that includes another primary African malaria vector, *An. arabiensis*. Additionally it contains several species, *i.e. An. melas, An. merus* and *An. bwambae*, that occasionally transmit malaria locally, but do not have wide enough distributions to be considered important vectors. More importantly for the purpose of the present study, the *An. gambiae* complex also contains the highly zoophilic *An. quadriannulatus A* and *An. quadriannulatus B*, which are never or rarely exposed to the human-limited *P. falciparum.*

In this study, we investigated patterns of polymorphism in three anti-malarial genes, *i.e. CEC1*, *GNBP-B1* and *LRIM1,* in six species of the *An. gambiae* complex. *CEC1* (ENSANGG00000009468, www.ensembl.org/Anopheles_gambiae) is a cecropin gene whose expression in *An. gambiae* is induced by infection with bacteria and *Plasmodium berghei* [16]. Additionally, genetically modified *An. gambiae* that express *CEC1* 24 hours after a blood meal, showed a 60% reduction in the number of *P. berghei* oocysts [4]. *GNBP-B1* (ENSANGG00000015205) is a pattern recognition receptor whose expression is strongly upregulated in response to infection with both *P. berghei* [17], and *P. falciparum* [5]. *LRIM1* (ENSANGG00000010552) is a leucine-rich repeat immune protein that is an important plasmodium antagonist. This protein is up-regulated in response to infection with *P. berghei,* and silencing of this gene increases oocyst load 3.6-fold [6]. Furthermore, this gene has been implicated in the melanization reaction of parasites [18]. We performed various tests for positive selection on these anti-malarial genes in the two main vectors, *An. gambiae* and *An. arabiensis,* to examine if these genes show signs of an adaptive response that may implicate them in the co-evolution of the mosquito vector and *Plasmodium* pathogen. Whereas no evidence for positive selection was found in *CEC1* and *GNBP-B1*, our results clearly indicate that *LRIM1* underwent an adaptive response in the *An. arabiensis* lineage. Additionally our data also indicate that *LRIM1* has introgressed from *An. arabiensis* into *An. gambiae.*

## Results

The complete *CEC1* gene, consisting of 177 bp of coding sequence and two introns comprising a combined 90 bp, was amplified. A total of 186 alleles of this gene were obtained from six species of the *An. gambiae* complex, 66 of which were unique (Table 1, genbank accession nos EU073463–EU073527). Although several polymorphisms were shared between species, none of the alleles were. For the coding region, *Dxy,* the average number of nucleotide substitutions between alleles in different species, ranged from 0.829 to 2.54 (per 100 bp). Very few fixed differences were present between species, and in most comparisons no fixed non-synonymous differences were found (Table 2). Not surprisingly therefore, none of the McDonald-Kreitman tests indicated an excess of non-synonymous fixed differences between species. In particular, no fixed amino-acid changes were observed between the non-vector species *An. quadriannulatus A* and the two major malaria vectors *An. gambiae* and *An. arabiensis*.

**Table 1:** Number of sampled alleles.

	*CEC-A*	*GNBP-B1*	*LRIM1*
*gam*	49 (22)	6 (6)	28 (26)
*ara*	25 (15)	8 (8)	36 (35)
*qua*	21 (10)	7 (7)	25 (14)
*mer*	19 (11)	6 (6)	14 (13)
*mel*	57 (6)	6 (6)	22 (13)
*bwa*	15 (2)	4 (4)	13 (7)

Number of unique alleles is between brackets.

*gam* = *An. gambiae*, *ara* = *An. arabiensis*, *qua* = *An. quadriannulatus A*, *mer* = *An. merus*, *mel = An. melas*, *bwa = An. bwambae.*

**Table 2:** MacDonald-Kreitman test on *CEC1.*

	Fixed		Polymorp.		
	S	NS	S	NS	*p-*value
*gam-ara*	2	0	6	6	n.s.
*gam-qua*	2	0	5	5	n.s.
*gam-mel*	0	0	4	6	-
*gam-mer*	2	0	3	6	n.s.
*gam-bwa*	2	1	4	5	n.s.
*ara-qua*	0	0	6	1	-
*ara-mel*	2	1	4	2	n.s.
*ara-mer*	2	0	3	2	n.s.
*ara-bwa*	0	1	4	1	n.s.
*qua-mel*	2	1	4	1	n.s.
*qua-mer*	2	0	3	1	n.s.
*qua-bwa*	0	1	4	0	n.s.
*mel-mer*	2	1	1	2	n.s.
*mel-bwa*	2	2	2	1	n.s.
*mer-bwa*	2	0	1	1	n.s.

S = synonymous, NS = non-synonymous. Species names are abbreviated.

For *GNBP-B1* a total of 38 alleles from six species, consisting of the complete 1188 bp coding sequence and 232 bp of intron sequence, were obtained (Table 1, genbank accession nos EU073426–EU073462). All of these alleles were unique, but some polymorphisms were shared between species. *Dxy* ranged from 0.723 to 2.5 (per 100 bp) for the coding region. Very few fixed replacement substitutions were observed between species (Table 3). Two of the McDonald-Kreitman tests were significant. However, both indicated an excess of non-synonymous polymorphisms, and in all comparisons the ratio of non-synonymous to synonymous substitutions was higher for polymorphisms than for fixed differences. Between the non-vector species *An. quadriannulatus A* and the malaria vectors *An. arabiensis* and *An. gambiae,* only a single replacement substitution was observed.

**Table 3:** MacDonald-Kreitman test on *GNBP-B1.*

	Fixed		Polymorp.		
	S	NS	S	NS	*p*-value
*gam-ara*	0	0	26	17	-
*gam-qua*	17	1	32	12	n.s.
*gam-mel*	13	0	20	12	n.s.
*gam-mer*	17	5	22	17	n.s.
*gam-bwa*	0	0	19	13	n.s.
*ara-qua*	14	1	28	11	n.s.
*ara-mel*	9	0	16	10	0.036
*ara-mer*	14	5	18	15	n.s.
*ara-bwa*	0	0	17	12	-
*qua-mel*	13	1	18	5	n.s.
*qua-mer*	18	3	20	10	n.s.
*qua-bwa*	0	0	15	3	-
*mel-mer*	14	5	8	9	n.s.
*mel-bwa*	13	0	10	7	0.010
*mer-bwa*	17	4	12	12	n.s.

S = synonymous, NS = non-synonymous. Species names are abbreviated.

For *LRIM1* we sequenced 858 bp that were thought to represent a single exon constituting the entire gene. However, in the most recent release of the Ensembl *An. gambiae* genome (release 45) the annotation of this gene was altered, and it is now thought that these 858 bp represent about half of the coding sequence of *LRIM1*. We obtained a total of 138 alleles from six species, of which 108 were unique (Table 1, genbank accession nos EU073528–EU073597). As in the other two genes investigated here, polymorphisms were shared between species, but alleles were not. *Dxy* ranged from 1.03 to 3.06 (per 100 bp) between species. In contrast to *CEC1* and *GNBP-B1* however, McDonald-Kreitman tests of positive selection indicated a significant excess of fixed non-synonymous differences between *An. arabiensis* and *An. quadriannulatus A, An. merus* as well as *An. bwambae* (Table 4). The fact that all three comparisons involve *An. arabiensis,* suggests this lineage underwent more non-synonymous substitutions than expected under a neutral model.

**Table 4:** MacDonald-Kreitman test on *LRIM1.*

	Fixed		Polymorp.		
	S	NS	S	NS	*p*-value
*gam-ara*	0	0	69	37	-
*gam-qua*	0	0	48	33	-
*gam-mel*	3	3	53	33	n.s.
*gam-mer*	2	4	56	35	n.s.
*gam-bwa*	0	0	50	30	-
*ara-qua*	3	8	36	22	0.047
*ara-mel*	5	7	38	23	n.s.
*ara-mer*	2	10	45	25	0.003
*ara-bwa*	2	7	39	25	0.037
*qua-mel*	8	8	14	14	n.s.
*qua-mer*	6	6	19	17	n.s.
*qua-bwa*	0	0	14	17	n.s.
*mel-mer*	7	10	23	17	n.s.
*mel-bwa*	7	7	19	17	n.s.
*mer-bwa*	4	5	24	20	n.s.

S = synonymous, NS = non-synonymous. Species names are abbreviated.

**Figure 1:**
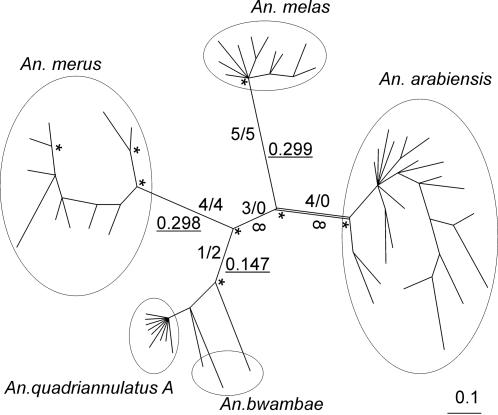
Bayesian tree (unrooted) of *LRIM1* from five species of the *An. gambiae* complex. Posterior probabilities ≥0.99 are indicated by *. Number of non-synonymous/synonymous substitutions are indicated above or on the left side of the branches. Estimated ω values are placed below or on the right side of the branches and are underlined. The foreground branch for the maximum likelihood tests of positive selection is indicated by a double line. For a more detailed phylogeny, including all posterior probabilities above 50% and sample names, see Figure S2 (supporting materials).

Surprisingly no fixed differences were present between *An. gambiae* and three other species; *An. arabiensis, An. bwambae* and *An*. *quadriannulatus A.* Our phylogenetic analysis showed that alleles from *An. gambiae* did not form a single cluster but were interspersed across the tree (Figure S1, supporting information). In particular, several “*arabiensis*-like” *An. gambiae* alleles clustered with *An. arabiensis*, far removed from the majority of *An. gambiae* sequences. When *An. gambiae* was removed from the analysis (Figure 1), *An. arabiensis, An. melas* and *An. merus* formed monophyletic groups with posterior probabilities of 0.99 and higher. *An. bwambae* formed a paraphyletic group containing the monophyletic *An. quadriannulatus A*. Since the *An. gambiae* alleles did not form a single cluster, it was not possible to test for positive selection along a single branch leading to this species. However, we did test for positive selection along the branch leading to the other major malaria vector, *An. arabiensis.* To increase the power of this analysis, the length of this branch, henceforth referred to as the foreground branch, was maximized by excluding all *An. gambiae* alleles from the analysis.

Due to the absence of synonymous substitutions the ω value, *i.e.* the ratio of non-synonymous to synonymous substitutions or dN/dS, along the foreground branch could not be estimated in our PAML analyses and is indicated as ∞ (Table 5). The branch test did not indicate that ω along the foreground branch is significantly larger than 1 (model 2 _free ω_ vs. model 2 _ω = 1_). However, a branch test did show that it is significantly larger than ω along the background branches (p = 0.011, model 2 _free ω_ vs. model 0). Values of ω along the branches leading to *An. melas, An. merus* and *An. bwambae*/*An. quadriannulatus A* were estimated to be 0.299, 0.298 and 0.147 respectively (Figure 1), indicating purifying selection along these lineages. This confirms that the excess of non-synonymous substitutions detected by the McDonald-Kreitman tests mainly occurred along the lineage leading to *An. arabiensis.* Interestingly, the branch separating *An. arabiensis* and *An. melas* from the other species also lacked synonymous, but not non-synonymous substitutions, possibly indicating some positive selection along this branch.

**Table 5:** Likelihood Ratio Test for positive selection on *LRIM1* in *An. arabiensis.*

Model	background ω	foreground ω^a^	ln	χ^2^-value	p-value^c^
*branch test (H1: foreground ω>background ω)*					
model 0	0.215	0.215	−2508.11		
model 2 _free ω_	0.204	∞^b^	−2504.88	6.46	0.011
*branch test (H1: foreground ω>1)*					
model 2 _ω = 1_	0.204	1	−2505.80		
model 2	0.204	∞^b^	−2504.88	1.84	0.175
*branch-site test*					
model A1	n.a.	n.a.	−2487.45		
model A	n.a.	n.a.	−2483.48	7.94	0.005

a foreground branch is branch leading to *An. arabiensis* (Figure 1).

b ω could not be estimated because the number of synonymous substitutions along foreground branch = 0.

c Based on χ^2^ distribution with df = 1.

The branch tests applied above use an ω value averaged across the entire gene. This severely diminishes the power of the analysis because at least some parts of each gene are expected to be under purifying selection. A more powerful test for positive selection is provided by branch-site models, which test if certain codons are under selection in the foreground branch [19]. When implemented on our data set (model A vs. model A1), the branch-site test provided strong support for positive selection in the *An. arabiensis* lineage with p = 0.005 (Table 5). According to the Bayes Empirical Bayes (BEB) analysis [20], amino-acid positions 108 and 236 are under positive selection (i.e. ω>1), with probabilities of 0.983 and 0.985, respectively. Position 235 has a high probability (0.933) of being under positive selection as well.

A total of four non-synonymous substitutions occurred along the foreground branch (Figure 1). Three of these non-synonymous substitutions are within two adjacent codons (235 and 236), with codon 236 having two replacement substitutions. That is, regardless of the order in which the two nucleotide substitutions in codon 236 occurred, two subsequent amino-acid changes were the result. Additionally, a replacement substitution at position 234 is fixed in *An. melas,* is at high frequency in *An. arabiensis* (0.83) and is also found in one of the “*arabiensis*-like” *An. gambiae* alleles.

**Figure 2:**
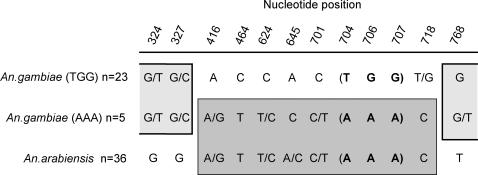
Shared polymorphism of *LRIM1* in *An. gambiae* (TGG), *An. gambiae* (AAA), and *An. arabiensis.* Only sites beyond position 324 that favor the clustering of *An. gambiae* (AAA) alleles with either *An. gambiae* (TGG) or *An. arabiensis* are included.

*An. arabiensis* is fixed for nucleotide A at sites 704, 706 and 707 (codons 235 and 236). With the exception of *An. gambiae,* all other species are fixed for nucleotides T, G and G at these respective positions. The few “*arabiensis*-like” *An. gambiae* alleles also have the (AAA) arrangement. Positions 416 through 718, a 302 bp stretch, cluster these *An. gambiae* (AAA) alleles with *An. arabiensis* (Figure 2). Only positions below 328 and above 767 contain polymorphisms that group some of the *An. gambiae* (AAA) alleles with the rest of *An. gambiae.* Additionally, no fixed differences were found between *An. gambiae* and *An. arabiensis* anywhere in this gene.

*LRIM1* is located inside the *2La* inversion. Therefore we determined the karyotype of our *An. gambiae* samples with respect to the *2La* arrangement. Out of 32 specimens from Cameroon, three were *2La/+* heterozygotes, all of which were also heterozygous for the (AAA)/(TGG) alleles. The karyotype of one (AAA)/(TGG) heterozygote was not clear, as it produced a second band of unexpected size. The remaining four (AAA)/(TGG) heterozygotes, as well as all (TGG) homozygotes, carried the *2L+/+* karyotype. These findings confirm that the (AAA) allele is at very high frequency or even fixed in the *2La* inversion in Cameroon, and is present at very low frequency in the *2L+* arrangement (≈7%).

To examine if *LRIM1* in *An. arabiensis* showed signs of a recent selective sweep, a HKA test was performed by comparing the polymorphism/fixed differences ratio of *LRIM1* to CEC1 and GNBP-B1. A selective sweep reduces the amount of standing genetic variation within a species, as indicated by a relatively low ratio. However, no significant differences were found between the genes, and in fact, this ratio was considerably higher for *LRIM1* (20/20.2) as compared to *CEC1* and *GNBP-B1* (17/28.1).

## Discussion

*CEC1* and *GNBP-B1* did not show any signs of positive selection, and in particular, showed little or no differentiation between malaria vectors and the non-vector species, indicating that these genes are largely subject to purifying selection. In two of the species comparisons *GNBP-B1* showed a significant excess of non-synonymous polymorphisms. Some cloning error is expected to be present in the *GNBP-B1* data set. Since a majority of possible mutations are non-synonymous, random errors will bias the observed number of non-synonymous polymorphisms upward. However, the number of PCR errors in the data is not nearly high enough to explain the difference. Therefore, most likely purifying selection is responsible, with numerous slightly deleterious substitutions present at low frequency in populations, but which are prevented from going to fixation. In contrast, *LRIM1*, a gene with no known homologue in other organisms, shows clear signs of positive selection in *An. arabiensis.*

As pointed out by MacDonald and Kreitman, an excess of non-synonymous fixed differences between species may result from a much smaller population size in the past [21]. This would have allowed slightly deleterious mutations to go to fixation by drift, whereas in the current larger population most of these are removed by purifying selection. Evidence for population expansion has been reported for both *An. gambiae* and *An. arabiensis* [22], [23]. It is unlikely however that this could explain the excess of non-synonymous substitutions observed in *LRIM1* in *An. arabiensis*. First of all, a demographic explanation should affect all genes. As noted before, *GNBP-B1* has a higher ratio of non-synonymous/synonymous polymorphisms than fixed differences in most or all populations, including *An. arabiensis*. This indicates the presence of a relatively large number of slightly deleterious alleles in this gene, few or none of which became fixed in ancestral populations. This is contrary to the demographic explanation. More importantly however, it is extremely unlikely that three out of four fixed amino acid changes would occur in two adjacent positions in a 285 amino acid protein, if the random process of genetic drift were responsible.

Polymorphisms are shared between species in all three genes we examined, and there is no doubt some of this is due to the retention of ancestral polymorphism. However, the pattern of polymorphism observed in *LRIM1* provides strong evidence that the presence of “*arabiensis*-like” alleles in *An. gambiae* is caused by introgression and not by the retention of ancestral polymorphism. It is unlikely that recombination would not have broken down a linkage group of at least 352 bp, if it were maintained in the population for long time. Furthermore, the introgression hypothesis is supported by the complete absence of fixed differences between these species anywhere in the *LRIM1* gene.

Based on the shared polymorphisms between *An. gambiae* (AAA) and *An. arabiensis* (positions 416, 624 and 701), introgression has occurred multiple times, after which, according to the shared polymorphism between the *An. gambiae* (AAA) and (TGG) alleles (positions 324 and 327), these alleles recombined between position 327 and 416. The introgression of *LRIM1* from *An. arabiensis* into *An. gambiae* is also consistent with previous studies that have shown that introgression between these two species has occurred in the past [24], [25]. Additionally, it has been shown through sequence analyses [25], as well as crossing experiments [26], that different chromosomes vary in their capacity for horizontal transfer between these two species. The *2^nd^* chromosome, on which *LRIM1* is located, has been shown to transfer most readily between *An. gambiae* and *An. arabiensis*. [26].

Mosquitoes, like other organisms, encounter numerous pathogens during their life cycle, all of which could potentially exert selection pressure on the immune system. In fact, molecules that play a role in host-parasite interactions are one of the main groups of proteins on which positive selection has been demonstrated [11]. No direct evidence is available to show that *LRIM1* in *An. arabiensis* has evolved in direct response to malaria infection, but our observation that this anti-malarial gene shows distinct signs of positive selection in a primary malaria vector, but not in a species that does not transmit *Plasmodium*, is intriguing. This suggests that the observed adaptive evolution of *LRIM1* may have been the result of the infection of *An. arabiensis* with *Plasmodium*, with at least two adjacent amino acids playing a crucial role in this adaptation. If this is true, variation for *Plasmodium* resistance should be present at *LRIM1* in *An. gambiae*.

*LRIM1* contains a leucine-rich repeat (LRR), which in other LRR genes is crucial for the three-dimensional structure by folding it into an arc [27]. Little is known about the structure of *LRIM1*, but the two neighboring adaptive amino acids (*i.e.* 235 and 236) are located well outside the leucine-rich repeat region (positions 30–160), suggesting that they could have a more specific function.

Although *LRIM1* is known to play a role in suppressing *P. berghei* infection in *An. gambiae* [6], a recent RNAi study failed to show an effect of *LRIM1* on *P. falciparum* infection in field-collected *An. gambiae* [28], while an effect on *P. berghei* infection was confirmed. This could be because the action of *LRIM1* in *An. gambiae* is specific against *P. berghei.* However, it is also possible that only some *LRIM1* alleles suppress infection with *P. falciparum,* and these may even be specific for certain *P*. *falciparum* strains. Another study demonstrated the existence of such genotype by genotype interactions between *P. falciparum* and *An. gambiae*, by showing that no single strain of *P. falciparum* was best at infecting all of a set of iso-female *An.gambiae* lines [29]. Additionally, *LRIM1* is located inside the *2La* inversion. While *An. arabiensis* is fixed for this *2La* arrangement, *An. gambiae* is *2La/+* polymorphic. Since *LRIM1* alleles introgressed from *An. arabiensis* into *An. gambiae,* we may expect that these *An. gambiae* (AAA) alleles are mostly found in the *2La* arrangement. Interestingly, the mosquitoes that failed to show an effect of *LRIM1* knockdown on *P. falciparum* infection [28] all carried the standard chromosome arrangement (*2L+*). Our molecular karyotyping of the *2La* inversion in our *An. gambiae* specimens shows that the (AAA) allele is indeed found at very high frequency in *2La* inversions, whereas it is present in very low frequency in *2L+* (≈7%).

Pathogen-host co-evolution has mainly been considered in terms of an evolutionary arms race [30]. Under this model, the host continuously evolves to limit infection with the pathogen, which in turns evolves to evade host defenses. This is expected to lead to repeated selective sweeps, which leave a signature in the selected genes in the form of a low level of standing genetic variation. A comparison of the polymorphism to divergence ratio in *LRIM1* vs *GNBP-B1*/*CEC1* did not show a relatively low level of genetic variation in *LRIM1* in *An. arabiensis.* In fact, the relative level of polymorphism was higher in this gene than in *CEC1* and *GNBP-B1*. Therefore we have no indication that *LRIM1* in *An. arabiensis* is currently involved in an evolutionary arms race. This also implies that possible selective sweeps occurred long enough ago to allow mutation to regenerate polymorphism.

The data presented here indicate that the anti-malarial gene *LRIM1* has undergone adaptive evolution in a primary malaria vector. This could be because this gene has evolved in response to *P. falciparum* infection in this species. If so, *LRIM1* is expected to play a role in the resistance of *An. arabiensis* against *P. falciparum.* So far the immune system of this mosquito species has not yet been investigated, and our data suggest the possibility that a knockdown of *LRIM1* will enhance infections of *P. falciparum* in *An. arabiensis.* If *LRIM1* did indeed evolve in response to *P. falciparum* infection in *An. arabiensis*, this gene also deserves further study in *An. gambiae*, in particular with respect to potential variation in resistance between the two major alleles found in this species.

## Materials and Methods

### Mosquito sampling

Adult females of *An. gambiae* were collected from the villages of Mbebé and Nyabessan, Cameroon in Dec. 2005. *An. gambiae* from Mali were collected from Banambani in 2000. Adult *An. arabiensis* females from Cameroon were collected from Kousseri in Dec 2005. Adult *An. melas* were collected in Ipono, Cameroon, Dec. 2005. Larvae of *An. gambiae, An. arabiensis* and *An. bwambae* from Bwamba county, Uganda (2004) were kindly provided by Ralph Harbach. DNA extractions of *An. merus* from Furvela, Mozambique (2001 and 2003) were kindly provided by David O'Brochta. *An. quadriannulatus A* from Kruger National Park, South Africa, were kindly provided by Anton Cornel. Sample sizes for each gene and species are represented in Table 1.

### DNA methods

DNA was extracted using the DNeasy tissue kit (Qiagen). Species and molecular form diagnostics were performed following Fanello *et al.* [31] and Besansky *et al.* [32]. All *An. gambiae* specimens belonged to the S molecular form. Molecular identification of *2La* karyotypes was performed following White *et al.* [33]. Primers to amplify *CEC1, GNBP-B1* and *LRIM1* were designed using Primer3 [34] based on the *An. gambiae* genome and anneal to the flanking or non-transcribed regions of the genes [3]. PCR of *CEC-A* and *LRIM1* was performed using Amplitaq Gold polymerase (Perkin Elmer) using respectively the following primer pairs CECin1 (GTTAGCAGAGCCGTCGTCTT)/CECin12 (ACAGTCGGTTCAAAGCGTTC) and LRIM1in6 (AGGTAACGGACAGCAGCCTA)/LRIM1in9 (GTCCGGTACTGCTCCTTGAG). The following program was used for PCR amplification of *CEC1* and *LRIM1*; 2 min at 94°, 35 cycles of 30 sec at 94°, 30 sec at 52° and 45 sec at 72°, followed by 20 min at 72°. PCR products were excised from an agarose gel and purified using the Gel Purification Kit (Qiagen) and submitted for direct sequencing. A subset of the sequences from individuals heterozygous for two or more positions were amplified again and cloned using the TOPO-TA cloning kit (Invitrogen). Individuals were selected for cloning such that all observed polymorphic sites were represented in the final data set. From each individual a single colony was sequenced. PCR of *GNBP-B1* was performed using Platinum High Fidelity Taq (Invitrogen) with the primer pair GNBPin1 (GTTTGGTAGGGGACGAATGA) /GNBPIN20 (GCGCTTTCAGTGGTTTGTTT) using the following program: 2 min at 94°, 35 cycles of 30 sec at 94°, 30 sec at 52° and 90 sec at 72°, followed by 20 min at 72°. Direct sequencing of the PCR product of *GNBP-B1* was not possible in many cases because of the presence of indels. Therefore, PCR products of this gene were cloned and sequenced as outlined above. However, nine sequences were produced through direct sequencing, allowing for an estimation of the PCR/cloning error. Sequencing was performed on an ABI 3730 Genetic Analyzer using Big Dye v 3.1 (Applied Biosystems )

### PCR error

Based on a comparison between direct sequencing and plasmid sequencing, the PCR/cloning error using Amplitaq Gold was estimated to be approximately 1.5 per 1000 bp. However, because all *LRIM1* and *CEC1* samples that were cloned were also sequenced directly, we were able to derive both alleles from each individual while removing PCR/cloning errors. The PCR error in the *GNBP-B1* sequences amplified using the proof-reading polymerase was estimated to be 0.625 per 1000 bp. Therefore, each 1188 bp *GNBP-B1* allele for which no direct sequence was available is expected to have an average of 0.74 errors.

### Data analysis

All sequences were aligned using MEGA3.1 [35] and alignments were improved manually. Introns were included in the phylogeny reconstructions. For all other analyses the coding region was used. *Dxy* values were calculated using DnaSP 4.0 [36]. This software was also used to perform McDonald-Kreitman tests [21], using Fisher's exact test. The McDonald-Kreitman test compares the dN/dS ratio between species to within species and is based on the idea that substitutions under positive selection will go to fixation rapidly, and are therefore rarely observed as polymorphisms. However, they are present as fixed differences between species and an excess of replacement fixed differences is therefore an indication of positive selection.

Since few or no fixed differences were observed in *CEC1* and *GNBP-B1*, subsequent analyses were limited to *LRIM1*. Aimed at reducing the computational effort, a reduced *LRIM1* data set, containing 70 sequences, was used for phylogenetic analyses and maximum likelihood tests of positive selection. This data set was compiled in such a way that at every observed polymorphism and fixed difference, *i.e.* the relevant information for tests for maximum likelihood tests of positive selection, was retained. This reduced data set was used to construct 50% majority-rule consensus trees with MrBayes 3.1.2 [37]. Modeltest 3.7 [38] was used to determine the most appropriate nucleotide substitution model for our data set.

Several *LRIM1* alleles from *An. gambiae* clustered within *An. arabiensis* (Figure S1 supp. mat.). Therefore, phylogeny reconstruction was also performed excluding *An. gambiae* sequences. This inferred tree was used for maximum likelihood tests of positive selection along the branch leading to *An. arabiensis* in PAML3.15 and to estimate ω (*i.e.* dN/dS) along the major branches of the tree. Under the neutral model the relative number of synonymous and non-synonymous substitutions is expected to be 1. Under positive selection, amino acid substitutions are favored and ω>1, whereas under purifying selection amino acid substitutions are prevented and ω<1. The *An. arabiensis* lineage was designated as the foreground branch, *i.e.* the branch of interest, and model 2 _free ω_ was compared to model 0 to test if ω along the foreground branch was significantly larger compared to the ω along the background branches, *i.e.* all other branches. Model 2 _ω = 1_, with the ω value fixed at 1 along foreground branch, was compared to model 2 _free ω_ to test if ω along the foreground branch was significantly larger than 1. Model 1 was used to estimate ω along the central branches of the tree (Figure 1) and to infer the number of substitutions along these branches of the phylogeny. As an additional test for positive selection along the foreground branch, we used the more powerful branch-site test 2 by comparing model A and Model A1 [19]. Bayes Empirical Bayes (BEB) analysis was used to identify positively selected codons in the foreground branch [20].

To test for a reduction in the polymorphism of *LRIM1* in *An. arabiensis,* an HKA test was performed in DnaSP 4.0, using eight *An. arabiensis* alleles for *CEC1* and *LRIM1*, as well as all eight *GNBP-B1* alleles from this species. The *CEC1* and *LRIM1* alleles were from the same individuals as the *GNBP-B1* sequences if possible, otherwise were randomly chosen from the same population. Seven *An. quadriannulatus A* alleles from each gene were used to calculate inter-specific divergence.

## Supporting Information

Figure S1 Bayesian tree (unrooted) of *LRIM1* from six species of the *An.gambiae* complex. Posterior probabilities are indicated along branches. *An. gambiae* samples and *An. arabiensis* samples from Uganda, Madagascar and Mali are indicated by UG, MAD, and MAL respectively, remaining samples of these two species are from Cameroon.
(1.19 MB TIF)

Figure S2 Bayesian tree (unrooted) of *LRIM1* in five species of the *An.gambiae* complex. Posterior probabilities are indicated along branches. *An. arabiensis* samples from Uganda, Madagascar and Mali are indicated by UG, MAD, and MAL respectively, with all remaining *An. arabiensis* samples originating from Cameroon.
(0.72 MB TIF)
