# Supplementary figures and images for: Patterns of Selection in Anti-Malarial Immune Genes in Malaria Vectors: Evidence for Adaptive Evolution in LRIM1 in Anopheles arabiensis
Source: PLoS One. 2007 Aug 29;2(8):e793. doi: 10.1371/journal.pone.0000793 (PMC1945087; doi:10.1371/journal.pone.0000793)

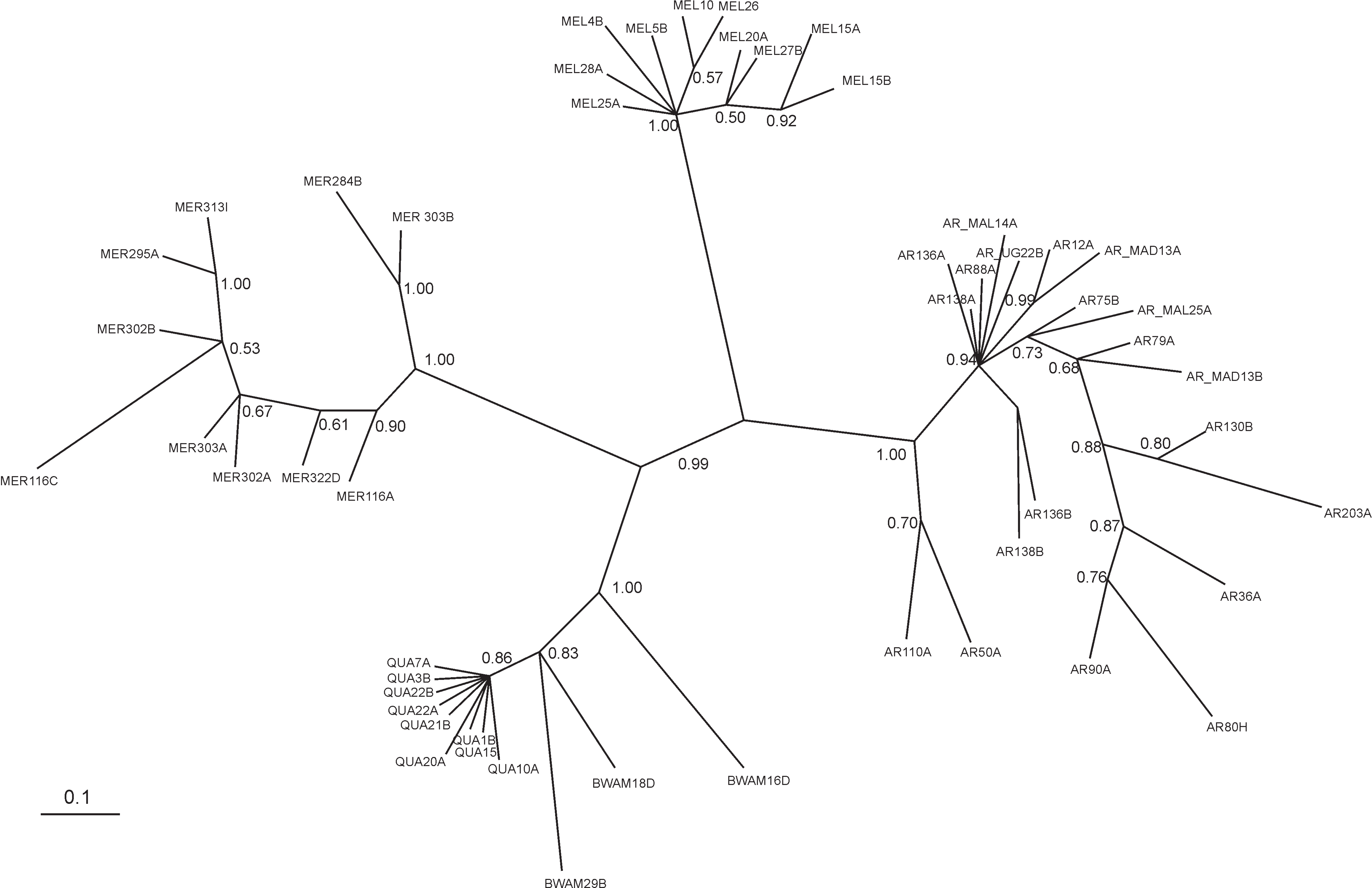

Supplement: Figure S2 — Bayesian tree (unrooted) of LRIM1 in five species of the An.gambiae complex. Posterior probabilities are indicated along branches. An. arabiensis samples from Uganda, Madagascar and Mali are indicated by UG, MAD, and MAL respectively, with all remaining An. arabiensis samples originating from Cameroon. (0.72 MB TIF) [file pone.0000793.s002.tif]
